# A Posterior Circulation Stroke Presenting with Isolated Truncal Ataxia

**DOI:** 10.7759/cureus.1709

**Published:** 2017-09-22

**Authors:** Daniel Migliaccio, Benjamin Lindquist

**Affiliations:** 1 Department of Emergency Medicine, University of North Carolina at Chapel Hill; 2 Department of Emergency Medicine, Stanford University School of Medicine

**Keywords:** stroke, emergency medicine, vertebral artery dissection

## Abstract

Vertebral artery dissection is an infrequent and often misdiagnosed cause of stroke. In this case report, we describe a patient with a posterior circulation stroke caused by a vertebral artery dissection, who presented to the emergency department with isolated truncal ataxia. This case emphasizes the importance of obtaining a thorough history and physical exam for all neurologic complaints, including a careful ambulation assessment.

## Introduction

Vertebral artery dissection (VAD) is a relatively infrequent cause of transient ischemic attacks (TIA) and strokes. Overall, VAD is thought to be the cause of approximately 2% of all ischemic strokes, with increased frequency in younger patients, accounting for approximately 20% of strokes in patients less than 45 years of age [1]. VAD has been associated with extreme or abrupt neck flexion/extension, such as during a sneeze, coughing fit, cervical manipulation, or sexual intercourse. Patients with VAD may present with vague symptoms, including headache and neck pain, with or without neurological findings, thus making it very difficult to diagnose. Neuroimaging using computed tomography angiography (CTA) or magnetic resonance imaging/magnetic resonance angiography (MRI/MRA) are the primary modalities for confirming suspected diagnosis. Treatment options include anticoagulation or antiplatelet therapy. In patients with signs of acute ischemia, intravenous thrombolysis or endovascular intervention may also be indicated [2-3]. 

## Case presentation

A 54-year-old female presented to the emergency department (ED) with gait instability that had commenced approximately 16 hours prior to arrival. Three days prior to presentation, she reported waking with a very mild posterior headache, a novel occurrence for the patient that persisted throughout her ED presentation. The night before arrival, she smoked marijuana, a familiar recreational activity, and consumed a single non-alcoholic beverage. While drinking her beverage, she recalled feeling "strange", and her husband noted that her gait had become abnormal. She was concerned that her drink had been altered or the bartender had mistakenly served her alcohol. The patient returned home to rest. She arrived at the ED the following morning with persisting headache and abnormal, unsteady gait. Prior to this incident, she had no history of similar symptoms. Her headache was described as very mild and diffuse without neck stiffness, photophobia or blurry vision. The patient did not report accompanying fever, chills, viral symptoms, weight loss, vomiting, head or neck trauma, chiropractic manipulation, or recent travel preceding or during the episode.

The patient’s medical history was significant for hypertension, hypothyroidism, chronic back pain, and remote poly-substance abuse. Her surgical history indicated a single cesarean section over 20 years ago. Her medications included hydrochlorothiazide, levothyroxine, and methadone. No known drug allergies were reported. Her social history was significant for smoking 2-6 cigarettes per day and usage of recreational marijuana, as well as heavy use of alcohol and methamphetamines in the remote past. With her husband out of the room, she expressed no marital concerns or domestic abuse.

On examination, the patient was afebrile, with a blood pressure of 135/75 mmHg, pulse of 80 beats/min, and respiratory rate of 18 breaths/min. On physical exam, she appeared generally well, but appropriately concerned about her condition. She did not smell of alcohol. She had no posterior neck tenderness or nuchal rigidity. Her neurological exam was notable for an appropriate mental status, and she spoke both fluently and conversantly while following commands without delay. The patient’s cranial nerve exam included equal pupils, round and reactive to light, and extra-ocular movements intact without nystagmus. Visual fields were full to confrontation. Her face was symmetric in strength and sensation with her palate elevated symmetrically, with the tongue extruding along the midline. Her motor and sensory exams were normal without pronator drift. Coordination examination indicated normal finger-to-nose and heel-to-shin movements, as well as normal rapid alternating movements. Her reflexes were 2+ and symmetric with toes downgoing bilaterally. However, her gait was wide-based with severe instability, preventing her from being able to walk without assistance. The patient’s Romberg test was positive with her eyes both open and closed. The remainder of her physical exam was unremarkable.

**Figure 1:**
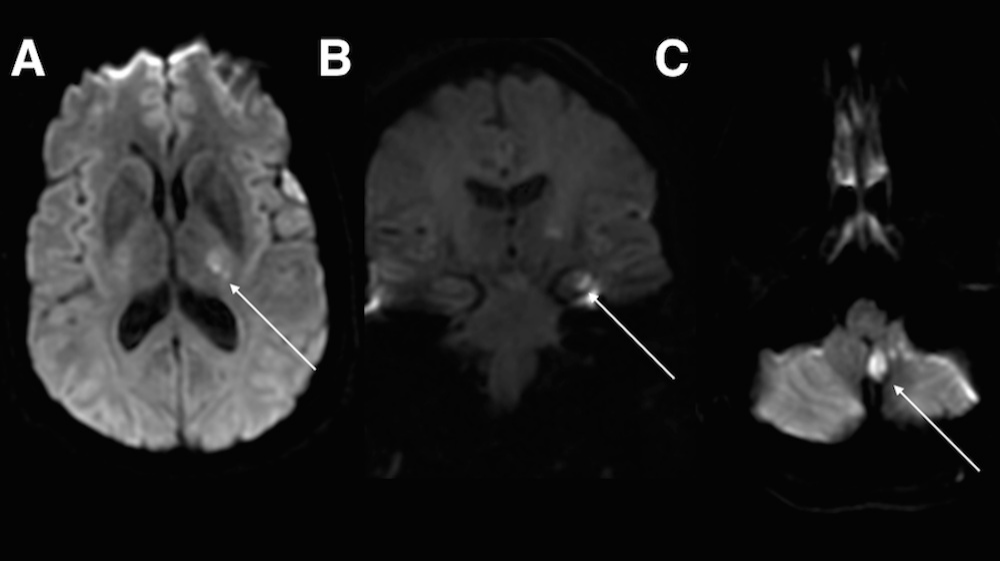
Magnetic resonance imaging (MRI) demonstrating acute infarct. Axial diffusion-weighted imaging (DWI) showing acute infarct in the left lateral thalamus (arrow - A), coronal DWI showing acute infarct in the left hippocampal tail (arrow - B) and axial DWI showing acute infarct in the left cerebellar tonsil (arrow - C).

**Figure 2:**
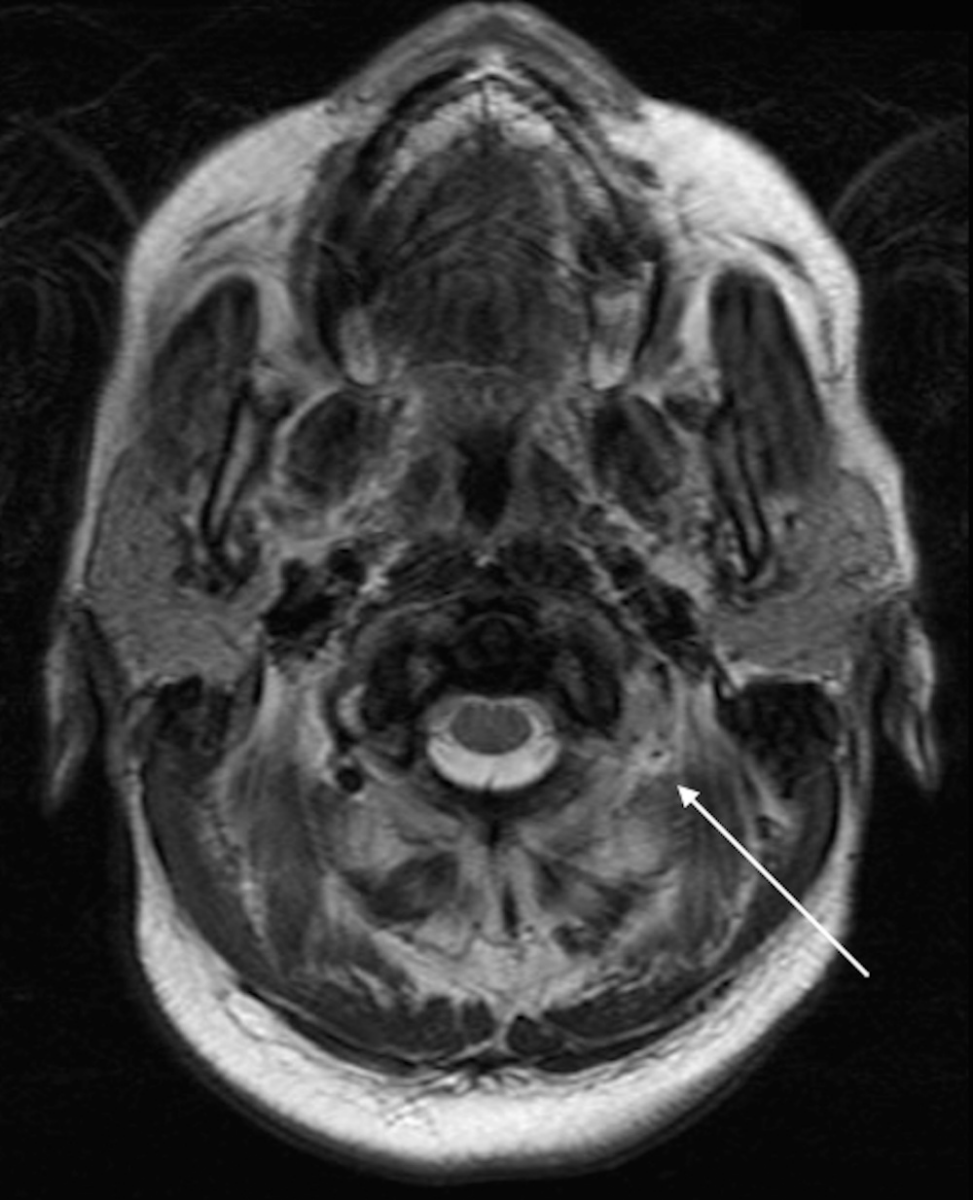
T2 magnetic resonance imaging (MRI) demonstrating flow void in the distal left vertebral artery (arrow) concerning for vertebral artery dissection.

Her initial laboratory results included a normal point-of-care glucose test, unremarkable complete blood count and basal metabolic panel, and negative urinalysis. The urine toxicology screen tested positive for only methadone and THC. Imaging included a normal chest radiograph. Neurology was consulted, and a brain MRI with and without contrast was ordered, revealing large acute infarcts in the left dorsal lateral thalamus, left hippocampal tail and body, and left cerebellar tonsil (Figure 1). Axial T2 MRI showed absence of flow in the left vertebral artery, likely resulting from vertebral artery dissection (Figure 2). The patient was admitted to the neurology service. The patient was subsequently started on aspirin and atorvastatin. There was no evidence of atrial arrhythmia on telemetry, or evidence of thrombus on echocardiogram. The patient received intensive physical and occupational therapy, and was subsequently discharged on the fourth day in the hospital with a walker to assist with her ambulation. At her two-month outpatient evaluation, she was continued on aspirin, high-dose statin, and blood pressure control. She was attempting to quit smoking. Her follow-up neurologic exam continued to show a hesitating and wide-based gait, as well as unstable heel-to-toe walking.

## Discussion

Vertebral artery dissection occurs secondary to tears in the intimal layer of the artery, allowing blood to track into the vessel wall and form a hematoma. If the hematoma that forms goes on to obstruct the vessel, it may block blood flow and lead to distal ischemia. In addition, the disruption of the endothelium can serve as a nidus for local thrombus formation, leading to possible embolization and infarction of distal brain parenchyma. VAD are classified as either spontaneous or traumatic. Approximately 10% of patients with spontaneous dissections are found to have structural defects of the arterial wall from connective tissue disorders, such as Ehlers-Danlos syndrome and Marfan’s syndrome. Dissection of the vertebral artery in these patients often occurs secondary to minimal rotational or flexion-extension injuries, or secondary to fractures extending into the transverse foramen. Up to 80% of patients with VAD present with neck pain and/or occipital headache [4]. In addition, they may have signs and symptoms consistent with posterior circulation ischemia, given the vascular territory supplied by the vertebral artery. In a retrospective study of all patients who sustained a VAD, transient ischemic attacks or stroke were presenting symptoms in 67% [1]. The most common symptoms on presentation are vertigo, ataxia, and diplopia. Patients may also less frequently present with focal neurological deficits, such as hemiparesis, dysarthria, and dysphagia.

In our case study, the patient presented to the ED with vague symptomatology that included isolated truncal ataxia. The differential diagnosis for ataxia includes cerebrovascular accident, intracranial hemorrhage, normal pressure hydrocephalus, Wernicke-Korsakov, and failed proprioception resulting from vitamin B12 deficiency or tabes dorsalis. However, ataxia symptoms may be ambiguous, and neurologic exams can be relatively normal. Our patient’s initial ED presentation directed providers and neurologic specialists to strongly consider intoxication as the cause of the patient’s symptoms, given her extensive poly-substance abuse history. However, adjunctive testing and neuroimaging were performed because the providers avoided two common pitfalls: discounting a patient’s concerns when they have a prior history of substance abuse; and failure to perform a comprehensive, ED-appropriate neurologic exam.

While intra-arterial angiography is considered the gold standard in the diagnosis of VAD, it has largely been replaced with non-invasive imaging modalities, such as MRI/MRA, CTA, and Doppler ultrasonography. Imaging in patients with VAD can demonstrate vertebral artery narrowing (51% of cases), as well as pseudo-aneurysm, double lumen, and intimal flap (22% each) [5]. A recent review comparing CTA and MRI/MRA revealed similar test characteristics between the two modalities, suggesting that neither test is superior to angiography [6]. Furthermore, MRI/MRA provided important complementary data, especially in regards to ischemic brain parenchyma and ischemic complications.

VAD prognosis depends on the extent of initial ischemic event and resulting collateral circulation. Resolution of the vertebral artery narrowing caused by the dissection, hematoma, or pseudo-aneurysm occurs in a majority of patients over time (90%). Approximately 75% of patients experience favorable functional outcome by six months in less severe ischemic attacks. Overall, the mortality rate from VAD is approximately 5%, and the risk of recurrent VAD is 1% annually [7].

Given that most cases of cerebrovascular dissection heal spontaneously, the goal of treatment is to protect at-risk brain tissue from future injury. There is no consensus on the best management of acute VAD. Anticoagulation, such as heparin followed by warfarin, is commonly prescribed; however, this regimen is contraindicated in cases of large infarcts with mass effect, or any infarct deemed high risk for hemorrhagic transformation. Likewise, antiplatelet agents may be recommended. Antiplatelets and anticoagulation in VAD have demonstrated equivalent outcomes with regards to morbidity and mortality, and similar side effects [8]. Two large randomized control trials found anticoagulation and antiplatelet agents were equivalent in their prevention of VAD-related stroke [9-10]. The use of thrombolytics is controversial, but may also be considered for patients presenting with stroke resulting from vertebral artery dissections. While not directly studied in VAD, a recent metanalysis of thrombolysis in ischemic stroke caused by cervical artery dissection (which includes both carotid and vertebral dissection-related stroke) found that thrombolysis in patients has comparable safety and functional outcomes to thrombolysis in patients with non-dissection-related strokes [2]. However, further randomized controlled trials (RCT) need to be performed to assess the safety and efficacy of thrombolytics in strokes caused solely by VAD, prior to routine use of thrombolysis in these patients. Also, there is no data to date comparing thrombolytics to other anticoagulation treatments in VAD-related strokes. Lastly, endovascular treatment with coiling is another treatment option for VAD; however, its use is limited to patients who discontinued medical therapy in order to prevent further thromboembolic complications, patients with enlarging pseudoaneurysms, and those with contraindications to anticoagulation. Research indicates that patients treated with endovascular methods had a low incidence of future stroke/TIA (0.27% per year), but increased incidence of complications (10.3%), and increased risk of mortality (3.4 %) [3]. 

## Conclusions

A patient presenting to the ED with truncal ataxia on ambulation and a history of poly-substance abuse was ultimately diagnosed with vertebral artery dissection, an uncommon cause of acute stroke. Identification of a relatively rare etiology of stroke, despite the patient’s vague and limited symptomology, reinforces the importance of ambulating all patients as part of a thorough neurological examination.
